# Complete Genome Characterization of Korean Porcine Deltacoronavirus Strain KOR/KNU14-04/2014

**DOI:** 10.1128/genomeA.01191-14

**Published:** 2014-11-26

**Authors:** Sunhee Lee, Changhee Lee

**Affiliations:** Animal Virology Laboratory, School of Life Sciences, BK21 Plus KNU Creative BioResearch Group, Kyungpook National University, Daegu, Republic of Korea

## Abstract

In April 2014, porcine deltacoronavirus (PDCoV) was first identified in feces from diarrheic piglets in South Korea and found to be closely related to other PDCoV strains. The complete genome of the Korean PDCoV strain, KOR/KNU14-04/2014, was sequenced and analyzed to characterize PDCoV circulating in South Korea.

## GENOME ANNOUNCEMENT

Coronaviruses are enveloped, single-stranded, positive-sense RNA viruses belonging to the family *Coronaviridae* of the order *Nidovirales*, which are further divided into 4 genera, *Alphacoronavirus*, *Betacoronavirus*, *Gammacoronavirus*, and *Deltacoronavirus* (1, 2). Viruses of the *Alphacoronavirus* and *Betacoronavirus* genera have been detected in swine (3). The fifth porcine coronavirus was identified in 2012 in an investigation to discover new deltacoronaviruses in mammals and birds from China (2). In February 2014, the presence of porcine deltacoronavirus (PDCoV) was first announced in Ohio, United States, and since then, this novel coronavirus has been reported in 17 U.S. states (4–7).

Since the discovery of PDCoV in China, we have conducted a surveillance study to identify the presence of PDCoV and tested a total of 108 fecal and 5 intestinal homogenate samples from pigs with diarrheal disease. These samples were initially negative for transmissible gastroenteritis virus (TGEV), porcine epidemic diarrhea virus (PEDV), and rotavirus, or positive for at least one of three viruses. Two fecal samples received from different pig farms in April and June of 2014, each of which had been positive for rotavirus and negative for all three viral pathogens, respectively, tested positive for PDCoV by reverse transcription (RT)-PCR followed by sequencing confirmation of PCR amplicons. To further investigate the genetic relationship of the Korean and previously reported Chinese and U.S. strains, the complete genome of a representative PDCoV strain, KNU14-04, was sequenced using rapid amplification of cDNA ends (RACE) and next-generation sequencing (NGS) as described previously (8–10). The KNU14-04 NGS reads were assembled using the full-length PDCoV reference genomes from GenBank (4, 5).

The complete genomic sequence of KNU14-04 is 25,422 nucleotides (nt) in length, excluding the 3′ poly(A) tail and consists of the 539-nt 5′ untranslated region (UTR), the 18,803-nt replicase gene (nt 540 to 11,414 for 1a and nt 11,414 to 19,342 for 1b), the 3,483-nt spike (S) gene (nt 19,324 to 22,806), the 252-nt envelope (E) gene (nt 22,800 to 23,051), the 654-nt membrane (M) gene (nt 23,044 to 23,697), the 285-nt nonstructural gene 6 (NS 6) (nt 23,697 to 23,981), the 1,029-nt nucleocapsid (N) gene (nt 24,002 to 25,030), the 603-nt NS7 gene (nt 24,096 to 24,698), and the 392-nt 3′ UTR.

The full-length PEDV genome of KNU14-04 has nucleotide identities of 98.8 to 99.0% and 99.6 to 99.8% to 2 Chinese strains (GenBank accession numbers JQ065042 and JQ065043) and 8 United States strains (accession numbers KJ462462, KJ481931, KJ567050, KJ569769, KJ601777, KJ601778, KJ601779, and KJ601780), respectively. Comparing the complete genome of KNU14-04 to the Chinese strain HKU15-155, a 3-nt insertion was observed in each of the S genes and the 3′ UTR of strain KNU14-04, which is also present in strain United States/IA/2014/8734 (4).

To our knowledge, this is the first time that the complete genome sequence of PDCoV from South Korea has been determined. Our sequence data will provide further insight into the understanding of the epidemiology and evolution of PDCoV in South Korea and facilitate investigations on the genetic diversity of PDCoV worldwide.

### Nucleotide sequence accession number.

These KOR/KNU14-04/2014 PDCoV sequence data have been deposited in GenBank under the accession no. KM820765.
